# Oolong tea consumption and its interactions with a novel composite index on esophageal squamous cell carcinoma

**DOI:** 10.1186/s12906-019-2770-7

**Published:** 2019-12-10

**Authors:** Shuang Liu, Zheng Lin, Liping Huang, Huilin Chen, Yanfang Liu, Fei He, Xiane Peng, Weilin Chen, Ruigang Huang, Wanting Lu, Huimin Yang, Zhisheng Xiang, Zhihui Zhang, Zhijian Hu

**Affiliations:** 1 Department of Epidemiology and Health Statistics, Fujian Medical University Fujian Provincial Key Laboratory of Environment Factors and Cancer, School of public Health, Fujian Medical University, Fuzhou, 350108 China; 2 Department of Radiation Oncology, Anxi County Hospital, Quanzhou, 352400 China; 3 Department of Radiation Oncology, Zhangzhou Affliated Hospital of Fujian Medical University, Zhangzhou, 363000 China; 4 Key Laboratory of Ministry of Education for Gastrointestinal Cancer, Fujian Medical University, Fuzhou, 350108 China; 5 Department of Epidemiology and Health Statistics, Fujian Provincial Key Laboratory of Environment Factors and Cancer, School of public Health, Key Laboratory of Ministry of Education for Gastrointestinal Cancer, Fujian Medical University, Fuzhou, 350108 China

**Keywords:** Oolong tea consumption, ESCC, The risk index, Case-control study

## Abstract

**Background:**

No previous study has investigated the association between oolong tea consumption and esophageal squamous cell carcinoma (ESCC), we aim to elucidate the association between oolong tea consumption and ESCC and its joint effects with a novel composite index.

**Methods:**

In a hospital-based case-control study, 646 cases of ESCC patients and 646 sex and age matched controls were recruited. A composite index was calculated to evaluate the role of demographic characteristics and life exposure factors in ESCC. Unconditional logistic regression was used to calculate the point estimates between oolong tea consumption and risk of ESCC.

**Results:**

No statistically significant association was found between oolong tea consumption and ESCC (OR = 1.39, 95% CI: 0.94–2.05). However, drinking hot oolong tea associated with increased risk of ESCC (OR = 1.60, 95% Cl: 1.06–2.41). Furthermore, drinking hot oolong tea increased ESCC risk in the high-risk group (composite index> 0.55) (OR = 3.14, 95% CI: 1.93–5.11), but not in the low-risk group (composite index≤0.55) (OR = 1.16, 95% CI: 0.74–1.83). Drinking warm oolong tea did not influence the risk of ESCC.

**Conclusions:**

No association between oolong tea consumption and risk of ESCC were found, however, drinking hot oolong tea significantly increased the risk of ESCC, especially in high-risk populations.

## Background

Esophageal cancer is one of the most prevalent malignancies throughout the world; it is the eighth most common cause of cancer and the sixth most common cause of cancer death worldwide [1]. Esophageal cancer is the fifth most common cause of cancer in China. The incidence rates (per 100,000) were 21.17 in the year 2012 [2]. Among the types of this cancer, approximately 90% of cases are esophageal squamous cell carcinoma (ESCC), and 70% of these occur in China [3].

Tea is the most popularly consumed beverage in the world, and its consumption has grown in approximately 30 countries. It is classified into 3 major types: green tea (nonfermented), oolong tea (half-fermented), and black tea (fermented) [4]. Tea constituents, including polyphenols (such as epigallocatechin-3 gallate, epigallocatechin), lipids, vitamins and minerals, may act at numerous points of carcinogenesis, including cancer cell growth, apoptosis and metastasis [5, 6]. Oolong tea is one of the special teas in Fujian [7]. and it is one of the most popular beverages in Asian countries, especially in China. The degree of fermentation used for tea varies from 10 to 100%, causing differences in the active metabolites of different types of tea [8]. However, the degree of fermentation had little effect on the polyphenol contents in oolong tea. When the degree of fermentation used for oolong tea varies from 25 to 70%, the corresponding polyphenol contents only changed from 20.41 to 23.65% [8, 9]. Although there are many epidemiological studies about the association between green tea consumption and esophageal cancer [5, 10–14], there has been no study of the potential association between oolong tea consumption and ESCC.

Demographic characteristics (such as marital status, education level and economic status) and life exposure factors (such as tobacco smoking, alcohol drinking, hot food, pickled food, dried food, fruits and vegetables) have been identified as the major protective factors or risk factors for ESCC in previous studies [15–17]. For demographic characteristics and life exposure factors, William B et al. [18] indicated that only a number of those factors assess the degree of susceptibility, it was found that most candidates differed from the least predisposed by as much as a 30-fold difference in risk. Therefore, it is necessary to evaluate these factors with comprehensive indicators. In ESCC, few studies have integrated these demographic characteristics and life exposure factors into a composite index, resulting in insufficient evaluation of the role of demographic characteristics and life exposure factors in ESCC.

The aim of the present study was to determine whether there is an association between oolong tea consumption and ESCC and its joint effects with a novel composite index.

## Methods

### Study population

From January 2010 to December 2015, a hospital-based case-control study was conducted in Fujian Province, China. A total of 830 cases of newly diagnosed primary ESCC were recruited from the Zhangzhou Affiliated Hospital of Fujian Medical University in Fujian. All patients were histopathologically confirmed by the World Health Organization classification of esophageal cancer (ICD-10, code-C15). Cases of second primary cancer or those with previous chemotherapy or radiotherapy were excluded. In total, 899 controls were randomly recruited from patients in the same hospital, these groups were (1) orthopedics patients; (2) neurological patients. Cases and controls were frequency-matched by sex and age (+ 5 years). Finally, a total of 646 cases and 646 controls were recruited into the study.

### Data collection

Structured epidemiological questionnaires were used for collecting information from cases and controls. Detailed information including demographic information, education level, economic status (the unit of household income is Chinese yuan), tea drinking, tobacco smoking, alcohol drinking and dietary factors were collected. Tea drinking was defined as consumption of at least 1 cup of tea per week for more than 6 months. Details of tea drinking habits included the type of tea (green tea, oolong tea, black tea and other tea), the frequency of tea consumption per week (never drinking, 1–5 times/week, ≥5 times/week) and the temperature of tea (drinking hot tea refers to the intake of high temperature tea within 5 min of steeping; drinking warm tea refers to the intake of tea 5 to 12 min after steeping). Written informed consent was obtained from participants.

### Statistical analysis

Demographic characteristics (such as marital status, education level, etc.) and life exposure factors (such as tobacco smoking, alcohol drinking, etc.) were compared with the chi-squared test. The association of tea drinking with ESCC was evaluated by using unconditional logistic regression. Odds ratios (ORs), and corresponding 95% confidence intervals (CIs) were used to express the strength of these associations. An OR of 1.0 indicates that the distribution of exposures among cases are the same as that of controls, i.e. the exposure is not associated with the risk of ESCC. An OR greater than 1.0 indicates that exposure is positively associated with the ESCC. An OR less than 1.0 indicates that exposure might be a protective factor against the disease. When the 95% CI of the OR does not include 1.0, there is a significant association between the exposure and ESCC risk.

Two models were used for the unconditional logistic regression. Sex, age, educational level, and household income were used for adjusting the association between tea drinking and ESCC in model-1. Tobacco smoking, alcohol drinking, hot food, hard food, pickled food, fried food and fruit consumption were included in model-2 additionally. The model fit performance was evaluated according to the Akaike information criterion (AIC), and model-2 had lower AIC than model-1, indicating a better fit of model-2 over model-1.

To further explore a hot tea drinking habit at a potential risk factor for ESCC, the population was divided into 3 categories, i.e., never drinks tea, warm tea drinker or hot tea drinker, and the ORs were calculated. Since it is well established that some demographic characteristics and life exposure factors are closely associated with ESCC risk, we synthesized a novel individual risk index by summing the products of statistically significant variables and corresponding multivariable-adjusted regression coefficients [19]. The population with risk indexes above the median values was coded as higher risk, while the others with indexes below the median were considered as lower risk. The higher the risk index score, the more risk factors for ESCC. A forest plot was applied to demonstrate the potential effect modification by several demographic characteristics and the risk index. I squares (I^2^) and corresponding Q tests (α = 0.10) were used for depicting heterogeneity. The data were analyzed by using the Stata 10 software program. All statistical tests were two-sided with the significance level at 5%.

## Results

The characteristics of cases and controls are shown in Table 1. Cases and controls had similar distributions of sex, age and eating fried food (*P* > 0.05). However, the education level, household income, tobacco smoking, alcohol drinking, and eating hot food, hard food, pickled food and fruits were significantly different between these two groups (*P* < 0.05).

**Table 1:** The characteristics of cases and controls

Variables	Controls [n(%)]	Cases [n(%)]	*χ*^*2*^	*P*
Sex			0.000	1.000
male	433(67.0)	433(67.0)		
female	213(33.0)	213(33.0)		
Age			3.802	0.149
< 50	87(13.5)	97(15.0)		
50~60	233(36.1)	258(39.9)		
≥ 60	326(50.5)	291(45.0)		
Education Level			101.492	< 0.001
≤ Primary	281(44.7)	462(72.6)		
≥ Junior	347(55.3)	174(27.4)		
Household income (yuan)			120.587	< 0.001
< 2000/month	305(49.8)	493(79.6)		
≥ 2000/month	308(50.2)	126(20.4)		
Ever smoking			14.984	< 0.001
No	300(47.5)	236(36.8)		
Yes	332(52.5)	406(63.2)		
Ever drinking			18.166	< 0.001
No	428(67.8)	361(56.2)		
Yes	203(32.2)	281(43.8)		
Hot food			86.588	< 0.001
No	367(58.0)	205(32.0)		
Yes	266(42.0)	435(68.0)		
Hard food			39.041	< 0.001
No	314(49.8)	207(32.5)		
Yes	317(50.2)	430(67.5)		
Pickled food			39.799	< 0.001
< 5times/week	333(52.6)	224(35.1)		
≥ 5times/week	300(47.4)	415(64.9)		
Fried food			0.607	0.436
< 5times/week	452(71.6)	445(69,6)		
≥ 5times/week	179(28.4)	179(30.4)		
Fruits			66.239	< 0.001
≥ 5times/week	399(63.1)	259(40.3)		
< 5times/week	233(36.9)	283(59.7)		

The relationships between tea drinking habits and ESCC are shown in Table 2. No statistically significant association was found between oolong tea consumption and ESCC (OR = 1.39, 95% CI: 0.94–2.05). Compared with those who never drink oolong tea, the OR (95% CI) was 2.23 (1.29, 3.86) for those who drink 1~5 times/week. Drinking hot oolong tea significantly increased the risk of ESCC with OR (95% Cl) of 1.60 (1.06, 2.41). Nevertheless, green tea consumption was associated with lower risk of ESCC (OR = 0.43, 95% CI: 0.26–0.71 Table 2). The inversely association with green tea drinking was also found in populations with ≥5 times/week consumption, OR (95% CI) was 0.40 (0.23, 0.69) (Table 2).

**Table 2:** Tea Drinking Habits and Odds Ratios (95%*Cl*) among Cases and Controls

Variables	Oolong tea drinking (*n* = 518)		Green tea drinking(*n* = 210)		Other tea drinking(*n* = 249)		Tea drinking(*n* = 1270)	
	OR^a^(95% CI)	OR^b^(95% CI)	OR^a^(95% CI)	OR^b^(95% CI)	OR^a^(95% CI)	OR^b^(95% CI)	OR^a^(95% CI)	OR^b^(95% CI)
Tea consumption								
No	1.00	1.00	1.00	1.00	1.00	1.00	1.00	1.00
Yes	1.64 (1.14,2.36)	1.39 (0.94,2.05)	0.43 (0.27,0.68)	0.43 (0.26,0.71)	1.07 (0.72,1.59)	0.91 (0.59,1.39)	1.12 (0.82,1.52)	0.92 (0.66,1.28)
Frequency of tea consumption								
Never drinking	1.00	1.00	1.00	1.00	1.00	1.00	1.00	1.00
1~5 times/week	2.51 (1.49,4,23)	2.23 (1.29,3.86)	1.31 (0.68.2.54)	1.36 (0.62,2.97)	1.05 (0.59,1.87)	1.12 (0.60,2.09)	1.48 (0.99,2.22)	1.35 (0.88,2.08)
≥ 5 times/week	1.52 (1.04,2.22)	1.28 (0.85,1.92)	0.38 (0.23,0.62)	0.40 (0.23,0.69)	1.08 (0.69,1.69)	0.83 (0.51,1.36)	1.00 **(**0.72**,**1.39**)**	0.78 (0.55,1.14)
Duration of tea consumption								
Never drinking	1.00	1.00	1.00	1.00	1.00	1.00	1.00	1.00
1~10 year	1.41 (0.79,2.52)	1.22 (0.66.2.26)	0.17 (0.06,0.46)	0.16 (0.05,0.52)	1.25 (0.55,2.08)	1.47 (0.61,3.55)	0.82 (0.52,1.30)	0.75 (0.46,1.23)
≥ 10 year	1.78 (1.20,2.63)	1.26 (0.68,2.34)	0.55 (0.34,0.90)	0.57 (0.33,0.98)	0.98 (0.64,1.52)	0.78 (0.48,1.25)	1.16 (0.84,1.61)	0.91 (0.64,1.30)
Tea temperature								
Never drinking	1.00	1.00	1.00	1.00	1.00	1.00	1.000	1.00
warm	0.75 (0.41,1.36)	0.89 (0.42,1.67)	0.29 (0.13,0.65)	0.40 (0.15,1.03)	0.73 (0.35,1.52)	0.84 (0.39,1.81)	0.55 (0.35,0.86)	0.71 (0.44,1.15)
hot	1.96 (1.34,2.87)	1.60 (1.06,2.41)	0.49 (0.30,0.80)	0.45 (0.26.0.77)	1.10 (0.71,1.68)	0.88 (0.55,1.41)	1.28 (0.93,1.76)	0.94 (0.67,1.34)

^a^ Adjusted for sex, age, education level, household income (Model-1)

^b^:Adjusted for tobacco smoking, alcohol drinking, hot food, hard food, pickled food, fired food, fruits and the variables in model 1(Model-2)

Table 3 shows the associations between demographic characteristics and life exposure factors and risk of ESCC. In terms of demographic characteristics, populations with an education level above primary and household income more than 2000 yuan/month were associated with reduced risk of ESCC, the ORs (95% CIs) were 0.33(0.22, 0.48) and 0.28(0.19, 0.41), respectively. In terms of life exposure factors, an increased risk of ESCC was found to be associated with eating hot food, eating hard food, frequently eating pickled food (≥5 times/week) and very low daily fruit consumption (≤ 5 times/week), the ORs (95% CIs) were 1.73(1.23, 2.43), 1.67(1.18, 2.37), 1.52 (1.09, 2.11) and 2.12 (1.52, 2.95), respectively. Next, we synthesized a novel individual risk index by summing the products of statistically significant variables and corresponding multivariable-adjusted regression coefficients, the risk index was classified into two categories according to the median of the population (low risk group ≤0.55, high risk group > 0.55).

**Table 3:** The risk score coefficients for ESCC

Variables	Controls[n(%)]	Cases[n(%)]	β	OR(95% CI)^a^	Risk score
Sex					
male	230(62.2)	321(66.5)			
female	140(37.8)	162(33.5)	−0.354	0.70 (0.41,1.21)	
Age	370(100)	483(100)	−0.016	0.98 (0.97,1.00)	
Education level					
≤ Primary	156(43.9)	336(70.6)			
≥ Junior	199(56.1)	140(29.4)	−1.125	0.33(0.22, 0.48)	−1.125
Household income (yuan)					
< 2000/month	184(52.4)	372(81.4)			
≥ 2000/month	167(47.6)	85(18.6)	−1.271	0.28(0.19,0.41)	−1.271
Ever smoking					
No	175(49.0)	184(38.4)			
Yes	182(51.0)	295(61.6)	0.193	1.21(0.74,1.20)	
Ever drinking					
No	236(66.3)	278(57.9)			
Yes	120(33.7)	202(42.1)	0.313	1.37(0.90,2.08)	
Hot food					
No	181(50.6)	160(33.5)			
Yes	177(49.4)	317(66.5)	0.546	1.73(1.23, 2.43)	0.546
Hard food					
No	162(45.3)	156(32.8)			
Yes	196(54.7)	319(67.2)	0.514	1.67(1.18, 2.37)	0.514
Pickled food					
< 5times/week	192(53.5)	171(35.8)			
≥ 5times/week	167(46.5)	306(64.2)	0.416	1.52 (1.09, 2.11)	0.416
Fired food					
< 5times/week	265(74.0)	338(70.9)			
≥ 5times/week	93(26.0)	139(29.1)	−0.012	0.99(0.68,1.44)	
Fruits					
≥ 5times/week	226(63.0)	185(38.6)			
< 5times/week	133(37.0)	294(61.4)	0.750	2.12 (1.52, 2.95)	0.750

^a^ Adjusted for sex, age, educational level, household income, tobacco smoking, alcohol drinking, hot food, hard food, pickled food, fired food and fruits

**Fig. 1:**
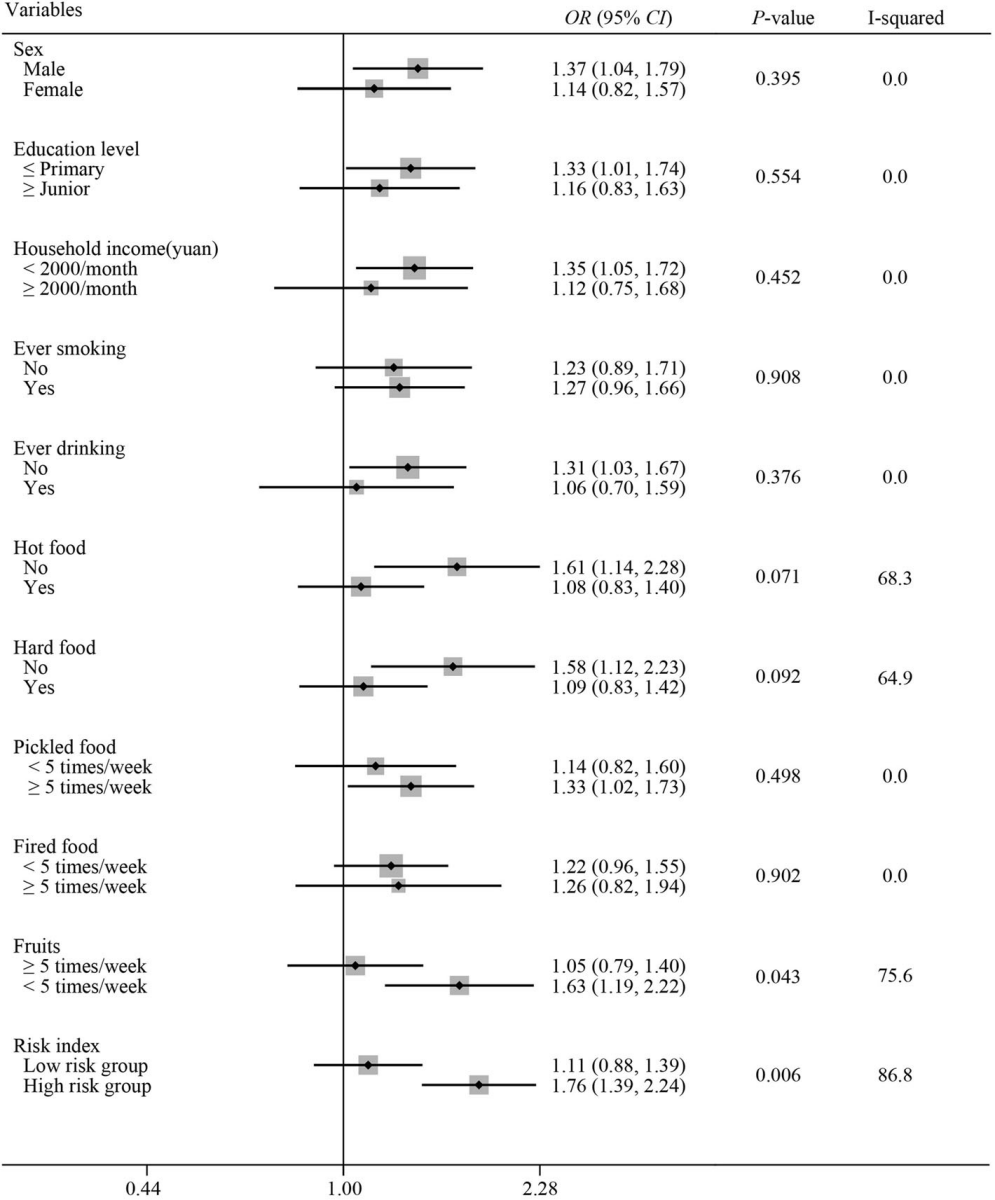
Multivariable odds ratios (odds) and 95% CI for the association between the oolong tea drinking temperature and ESCC risk across strata of various factors

The associations between the oolong tea drinking temperature and ESCC risk stratified by demographic characteristics and life exposure factors are observed in Fig. 1. An increased ESCC risk associated with oolong tea drinking temperature was detected in male, populations with household income less than 2000 yuan/month, education level not above primary, frequently eating pickled food (≥5 times/week), very low daily fruit consumption (≤ 5 times/week), but not in populations with histories of alcohol consumption, hot food or hard food intake. There was a multiplicative interaction between the temperature of oolong consumption and the risk index. The multiplicative OR (95% CI) was 1.59(1.14, 2.21), *P* = 0.006 (data not shown). Hot oolong tea increased the risk of ESCC only in those with high-risk indexes (OR = 1.76, 95% CI: 1.39–2.24).

The combined effect of the temperature of oolong tea drinking and the risk index group is presented in Table 4. There were no significant associations between warm oolong tea drinking and ESCC risk in the high-risk group and the low-risk group. Compared with those who did not drink warm oolong tea, odd ratios (95% CIs) were 0.70 (0.28, 1.74) for the population who drank warm oolong tea in the high-risk group and 0.63 (0.30, 1.30) for those who drank warm oolong tea in the low-risk group. Drinking hot oolong tea increased ESCC risk in the high-risk group (OR *=* 3.14, 95% CI:1.93*–*5.11). In the high-risk group, the frequency of hot oolong tea consumption 1~5 times/week and over 5 times/week significantly increased the risk of ESCC with OR (95% CIs) of 6.63 (2.26, 19.44) and 2.71 (1.63, 4.50), respectively, compared with never drinking. The duration of hot oolong consumption was significantly associated with risk of ESCC in the high-risk group. The OR was 3.16 (95% CI:1.15–8.70) for those who drank hot oolong tea 1~10 years and 3.46 (95% CI:2.03–5.91) for those who drank hot oolong tea for more than 10 years, but no significant association was found between drinking hot oolong tea and ESCC risk in the low-risk group.

**Table 4:** The combined effect of the temperature of oolong tea drinking and the risk index group

Risk index group	Variables	Drinking warm oolong tea	Drinking hot oolong tea
Low index group	Tea consumption		
	No	1.00	1.00
	Yes	0.63(0.30,1.30)	1.16(0.74,1.83)
	Frequency of tea consumption		
	Never drinking	1.00	1.00
	1~5times/week	1.21(0.36,4.01)	1.37(0.61,3.10)
	≥ 5 times/week	0.48(0.20,1.14)	1.16(0.73,1.86)
	Duration of tea consumption		
	Never drinking	1.00	1.00
	1~10 year	0.63(0.16,2.50)	0.94(0.40,2.20)
	≥ 10 year	1.04(0.23,4.84)	1.24(0.78,1.99)
High index group	Tea consumption		
	No	1.00	1.00
	Yes	0.70(0.28,1.74)	3.14(1.93,5.11)
	Frequency of tea consumption		
	Never drinking	1.00	1.00
	< 5times/week	0.38(0.45,32.26)	6.63(2.26,19.44)
	≥ 5 times/week	0.42(0.14,1.24)	2.71(1.63,4.50)
	Duration of tea consumption		
	Never drinking	1.00	1.00
	1~10 year	0.42(0.07,2.59)	3.16(1.15,8.70)
	≥ 10 year	0.63(0.20,2.05)	3.46(2.03,5.91)

## Discussion

This hospital-based case-control study was conducted in southeast China to illuminate the association between oolong tea consumption and ESCC and its joint effects with a composite index. In the present study, no association between oolong tea consumption and risk of ESCC were found. Drinking hot oolong tea significantly increases the risk of ESCC, especially for high-risk populations.

Some epidemiological studies have associated the consumption of tea with a lower risk of several types of cancers, including lung, gastric, esophageal and oral cavity cancers [13, 20–22]. Nevertheless, most studies focused on the association of green tea consumption and the risk of cancer. The chemical composition of green tea is complex and includes polyphenols, alkaloids (caffeine and theophylline) and other undefined compounds. Among these, the major composition is tea polyphenols, including epigallocatechin-3-gallate, epigallocatechin and epicatechin gallate [23–25]. Studies demonstrated that the mechanisms of action of tea polyphenols include induction of apoptosis, inhibition of cell proliferation and inhibition of tumor angiogenesis [24]. In the present study, we found that green tea consumption was associated with lower risk of ESCC. Our results were consistent with the previous studies.

Oolong tea is partially fermented, and its composition is similar to that of green tea. We suspected that oolong tea has similar effects to green tea, which can reduce the risk of ESCC. Nevertheless, we did not find the association between oolong consumption and ESCC risk, which may be explained by the lower tea polyphenol content of oolong tea relative to that of green tea and the lower antioxidant activity of oolong tea compared with that of green tea [26, 27].

In this study, we also found that drinking hot oolong tea significantly increases the risk of ESCC. The underlying mechanism is that chronic thermal irritation of the esophageal mucosa might stimulate the endogenous formation of reactive nitrogen species, which may directly or indirectly induce carcinogenesis [10, 28]. It has also been hypothesized that repeated thermal injury may impair the barrier function of the esophageal epithelium, therefore, making it more vulnerable to the damage by intraluminal carcinogens [29]. Farhad et al. [30] had reported that hot tea drinking increased the risk of esophageal cancer (OR = 2.07, 95% CI: 1.28–3.35), and such risk increased for very hot tea drinkers (OR = 8.16, 95% CI = 3.93–16.0). Chen et al. [10] showed that there was a significant protective effect of green tea on esophageal cancer with low temperature tea (OR = 0.79, 95% CI: 0.29–0.97), but not for 70–79 °C and above 80 °C drinkers, with ORs and 95% CIs 2.21(1.57–5.53) and 4.74(2.67–10.51), respectively. Another study provides quantitative confirmation that “very hot” drinking temperatures are probably causally related to esophageal cancer, whereas low temperature beverages are not [31].

Does hot oolong tea drinking cause changes in oolong tea active components? Study indicated that the chemical properties of tea polyphenols changed little when heated for 60 min and 90 min below 140 °C [32]. When preparing oolong tea infusion, the boiling water temperature for making oolong tea is 100 °C. Besides, the contact time between boiling water and oolong tea is short (less than 30 s). Therefore, in the process of making oolong tea, the content of polyphenols in oolong tea is relatively stable. Drinking hot oolong tea significantly increases the risk of ESCC, the potential mechanism is that the thermal stimulation of hot oolong tea drinking, not the action of oolong active components.

Stratified by demographic characteristics and life exposure factors, we found the association between hot oolong tea drinking and ESCC risk could be modified by sex, household income, education level, pickled food and fruit consumption. Pickled food contained N-nitroso compounds, which had the potential to induce the occurrence of EC [33]. Thermal injury of hot oolong tea drinking may impair the barrier function of the esophageal epithelium, making it more vulnerable to the damage by N-nitroso compounds. In contrast, there is overwhelming evidence that fruits were inversely associated with ESCC [34], which may attribute to their contents of carotenoids, vitamin A, vitamin C, and other antioxidants [35]. In the previous study, a similar effect was also reported by a cohort study that showed high temperature tea drinking and esophageal cancer risk was dependent on smoking and alcohol drinking; there was no such association in the absence of both habits [36]. Although some previous studies have discussed the roles of demographic characteristics and life exposure factors in ESCC, few have clarified the combined effect of these factors. To overcome this deficiency, we synthesized a novel individual risk index in this study. We found that drinking hot oolong tea increased ESCC risk only in the high-risk group, there was no statistically significant association between drinking warm oolong tea and the risk of ESCC in either the high-risk group or the low-risk group. The result showed that the hot oolong tea drinking and high-risk index might interact with each other and synergistically increase the risk of ESCC. We suggest that abstaining from hot oolong tea consumption might be beneficial for preventing ESCC in high-risk populations.

Several limitations should be considered in our study. First, selection bias may exist in any hospital-based case-control study. However, all subjects were recruited from the same hospital according to strict criteria, which may minimize the selection bias. The study data were obtained from interviews and might lead to recall bias. However, the definitions of variables were clearly given in our survey. As oolong tea consumption is a long-term personal habit, recall bias is relatively low. In addition, we used well-trained investigators in data collection to avoid information bias. Finally, although the fermentation time of oolong tea in different types, the Tie Guanyin of this study mainly comes from Anxi County (Fujian Province, China), which makes little variability in constituents of different oolong teas.

## Conclusions

No statistically significant association between oolong tea consumption and ESCC, but drinking hot oolong tea significantly increased the risk of ESCC, especially for high-risk populations. Our study suggests that abstaining from hot oolong tea consumption might be beneficial for preventing ESCC in high-risk populations.

## Data Availability

The datasets used and/or analyzed during the current study are available from the corresponding author on reasonable request.

## Abbreviations

95% CIs: Corresponding 95% confidence intervals
AIC: Akaike information criterion
ESCC: Esophageal Squamous Cell Carcinoma
ORs: Odds ratios
